# Manipulation of the Magnetic Properties of Janus WSSe Monolayer by the Adsorption of Transition Metal Atoms

**DOI:** 10.1186/s11671-021-03560-9

**Published:** 2021-06-10

**Authors:** Kai Chen, Weiqing Tang, Mingming Fu, Xu Li, Congming Ke, Yaping Wu, Zhiming Wu, Junyong Kang

**Affiliations:** 1 Department of Physics, OSED, Fujian Provincial Key Laboratory of Semiconductors Materials and Applications, Xiamen University, Xiamen, 361005 People’s Republic of China; 2 National Laboratory of Solid State Microstructures, Nanjing University, Nanjing, 210093 People’s Republic of China

**Keywords:** Magnetic anisotropy, First-principles calculations, Janus TMDCs, Surface adsorption

## Abstract

Two-dimensional Janus materials have great potential for the applications in spintronic devices due to their particular structures and novel characteristics. However, they are usually non-magnetic in nature. Here, different transition metals (TMs: Co, Fe, Mn, Cr, and V) adsorbed WSSe frameworks are constructed, and their structures and magnetic properties are comprehensively investigated by first-principles calculations. The results show that the top of W atom is the most stable absorption site for all the TM atoms, and all the systems exhibit magnetism. Moreover, their magnetic properties significantly depend on the adsorbed elements and the adsorbent chalcogens. A maximal total magnetic moment of 6 μB is obtained in the Cr-adsorbed system. The induced magnetism from S-surface-adsorption is always stronger than that for the Se-surface-adsorption due to its larger electrostatic potential. Interestingly, the easy magnetization axis in the Fe-adsorbed system switches from the in-plane to the out-of-plane when the adsorption surface changes from Se to S surface. The mechanism is analyzed in detail by Fe-3d orbital-decomposed density of states. This work provides a guidance for the modification of magnetism in low-dimensional systems.

## Introduction

Spintronics is an emerging technology exploiting the spin degree of freedom and holds great promise for next-generational devices with high speed and low power consumption [1–4]. Since the discovery of mechanically exfoliated graphene in 2004, there sets off a research boom on spin-electronic devices based on two-dimensional (2D) materials, especially on 2D graphene owing to its long spin diffusion length and coherent time [5–7]. However, graphene possesses zero band gap, limiting its development in optoelectronic devices [5]. Recently, transition metal dichalcogenides (TMDCs) are considered as promising candidates for optoelectronic applications due to the rich variety of materials and the tunable bandgap [8–11]. They generally exhibit a sandwich structure with the form of X–M–X (MX_2_, where M and X represent transition metal and chalcogen element, respectively), such as WS_2_ and WSe_2_, whose upper and lower layers have the same element. Interestingly, a new type of TMDCs, namely Janus structure of X–M–Y (X and Y represent different chalcogen elements, respectively) [12–14], shows many new features owing to the particular structures, such as strong Rashba spin–orbit coupling (SOC) effect [15, 16], large valley splitting [17, 18], strong piezoelectric effect [19], and so on. For example, Yao et al. reported that the Rashba coefficient of single-layer WSeTe is up to 0.92 eVÅ [15], which is even higher than that in traditional semiconductor heterojunctions of InGaAs/InAlAs [20] and LaAlO_3_/SrTiO_3_ [21]. Zhou et al. predicted that a large valley splitting of about 410 meV could be produced in Janus WSSe monolayer by the coupling with a MnO substrate [17].

Despite of the above-mentioned excellent properties, Janus TMDCs are intrinsically non-magnetic, which hinders its further application in spin-electronic devices. Up to now, the design and manipulation of low-dimensional magnetic materials is a major challenge. The previous research has introduced magnetism in conventional TMDCs through various approaches including the adsorption or doping of transition metal (TM) atom [22–25], the introduction of defects, chirality and edge structure [26–29], etc. Although it is predicted that the substitution of 3d-TM atoms can induce magnetism and modify the band structures in Janus MoSSe monolayer [30], such method is experimentally difficult to implement. In comparison, the surface atom adsorption is an effective and convenient way to tailor the physical properties of 2D materials. However, how the adsorbed atoms modify the electronic structure and physical properties of Janus TMDCs is rarely reported. The mechanism of magnetic regulation in Janus TMDCs remains unclear.

In this work, we construct different TMs (Co, Fe, Mn, Cr, and V) adsorbed WSSe frameworks, and comprehensively study their structures and magnetic properties by using first-principles calculations, especially their magnetic anisotropy energies (MAEs) at different adsorption surfaces. Their stable structures are identified by the calculated total energies, and their magnetic properties are analyzed by the total density of states (DOS) and differential charge densities. It is found that the magnetic moment and easy magnetization axis significantly depend on the adsorbed elements and the adsorbent chalcogens. In the case of Fe adsorption, the easy magnetization axis of the system can be switched from in-plane (Se side) to out-of-plane direction (S side). The physical mechanism of magnetic anisotropy is further analyzed by Fe-3d orbital-decomposed DOS.

## Computational Methods

All calculations are performed by density functional theory based on periodic boundary conditions with spin-polarized, as implemented in Vienna ab initio simulation package (VASP) code [31]. The generalized gradient approximation (GGA) with the Perdew–Burke–Emzerhof (PBE) description is adopted for the exchange–correlation potential [32, 33]. In order to rule out the interaction between TM atoms, a 4 × 4 × 1 supercell is selected. A 15 Å vacuum layer is used to eliminate the interlayer interaction and the periodic image. The interlayer vdW interaction is described by using the DFT-D2 method [34]. All structures are fully relaxed until the force and the total energy reach the convergence criterion, where the convergence values are set to 10^–6^ eV and 0.01 eV, respectively. The Brillouin zone is sampled with 7 × 7 × 1 and 11 × 11 × 1 dense mesh by using Gamma-centered Monkhorst–Pack grid in structural optimization and self-consistent calculation, respectively. The cutoff energy of the plane wave expansion is optimized to 500 eV, which ensures the convergence of the system. The MAE is calculated by taking the difference between the total energies as the magnetization oriented along the in-plane [100] and out-of-plane [001] directions: MAE = *E*_in_ – *E*_out_, SOC is considered in the calculations [35, 36].

## Results and Discussion

**Fig. 1:**
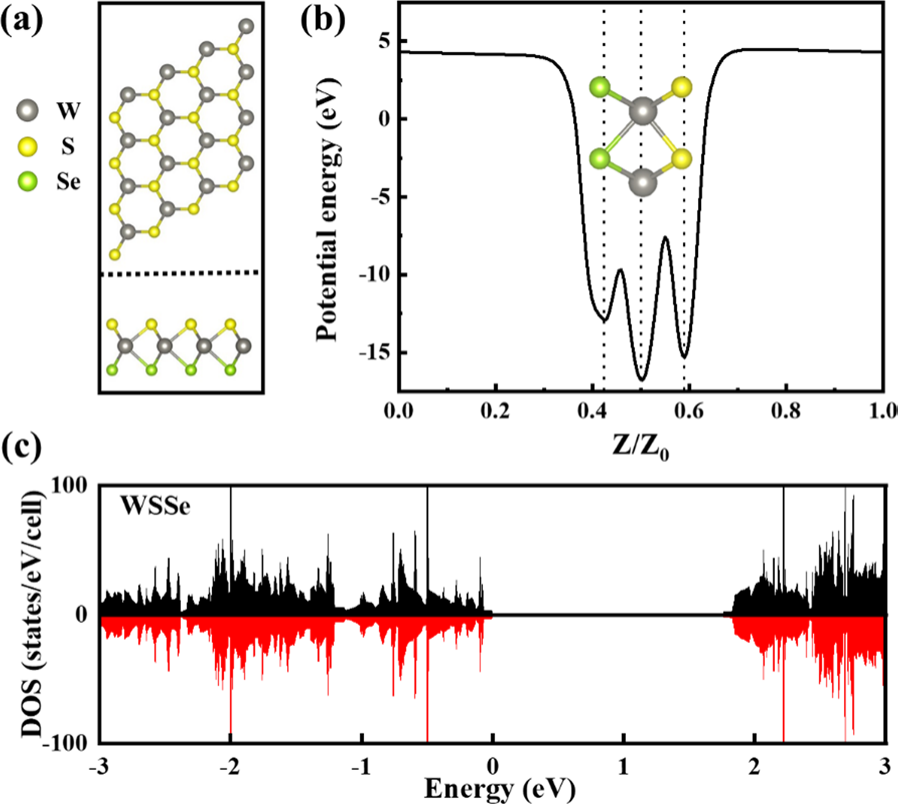
**a** Top view and side views of Janus WSSe monolayer. **b** The average in-plane electrostatic potential distribution of WSSe monolayer. **c** Total DOS of primitive Janus WSSe monolayer

To simulate the adsorption of TM atoms on Janus WSSe monolayer, we first construct a monolayer WSSe supercell consisting of 48 atoms, as shown in Fig. 1a. Pristine Janus WSSe monolayer possesses a spatial C_3v_ symmetry and exhibits the sandwich structure with one layer of S atoms, one layer of W atoms, and one layer of Se atoms. The monolayer thickness is calculated to be 3.35 Å. The planar projection shows an ideal hexagonal honeycomb structure with a lattice constant of 3.24 Å. The bond lengths of W-S (d_W-S_) and W-Se (d_W-Se_) are 2.42 Å and 2.54 Å, respectively, and the bond angle θ_S-W-Se_ is 81.76°, which are consistent with the previous reports [37]. Figure 1b shows the planar average electrostatic potential energy of the monolayer WSSe, where *Z*_0_ is the thickness of the unit cell, *Z* is a coordinate variable, and *Z*/*Z*_0_ means the relative position in the unit cell. As expected, the broken mirror symmetry along the *Z* direction results in the different potential energies on the S and Se surfaces, and the S surface has the larger electrostatic potential. Meanwhile, we also calculated the spin-resolved DOS of Janus WSSe monolayer. As shown in Fig. 1c, the DOS for the spin-up and spin-down channels are symmetrically distributed, indicating that the ground state is non-magnetic. It can also be seen that the band gap of Janus WSSe monolayer is about 1.7 eV, which is between that of WS_2_ [38] and WSe_2_ [39].

**Fig. 2:**
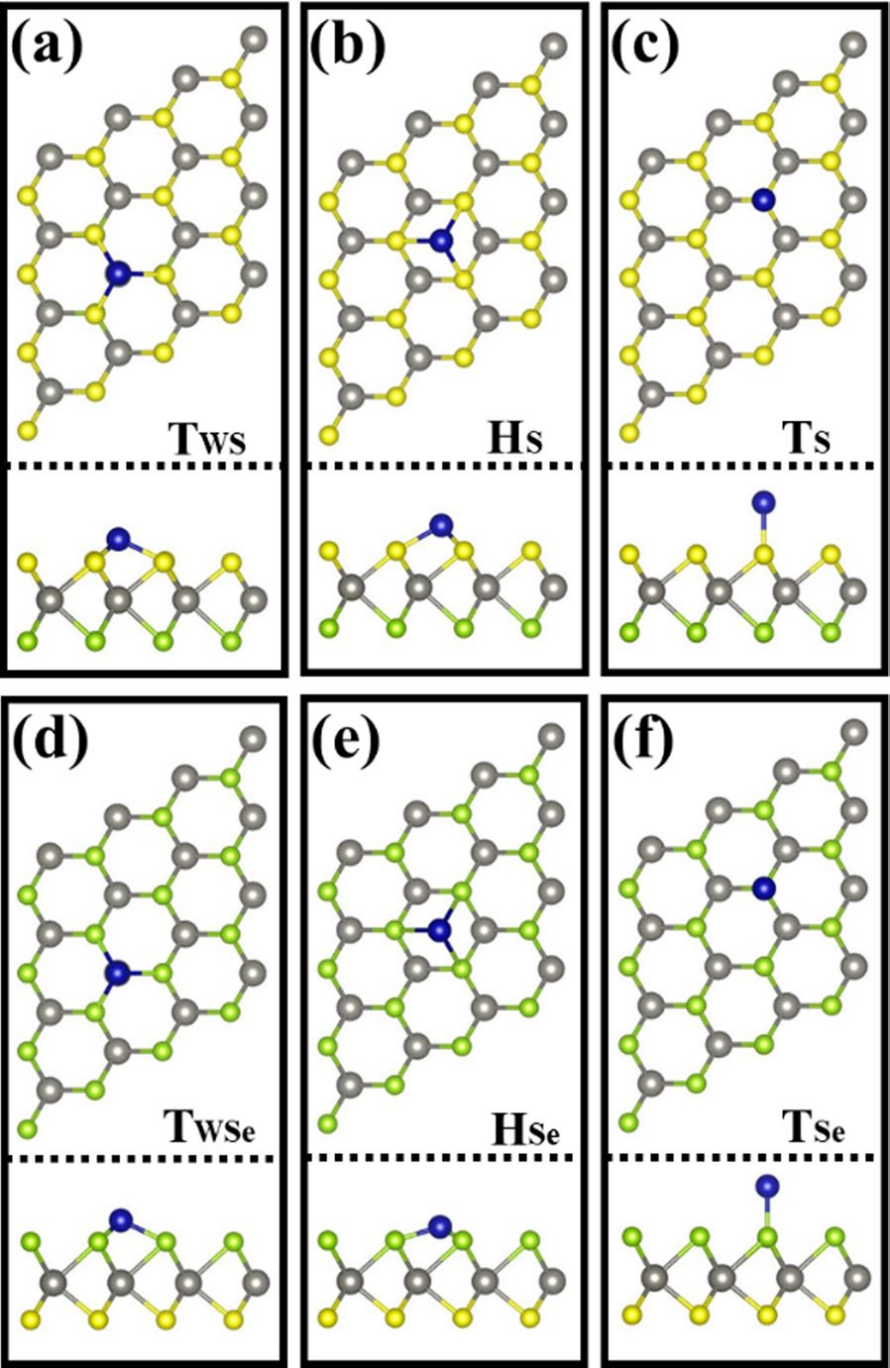
Top view and side views of different configurations. **a**, **d** TM atom locates on the top of W atom; **b**, **e** TM atom locates on the hollow site; **c**, **f** TM atom locates on the top of S(Se) atom

To induce magnetism in Janus WSSe, five different kinds of 3d-TM atoms (Co, Fe, Mn, Cr, and V) were adopted to adsorb on the surface of monolayer WSSe. On account of the structural symmetry, three possible adsorption sites are considered for atom adsorption on either the S or Se layer. As shown in Fig. 2, the three cases are on the top of W atom (labeled as T_WS_ or T_WSe_), on the hollow of hexagonal ring (labeled as H_S_ or H_Se_), and on the top of S (Se) atom (labeled as T_S_ or T_Se_). The total energies for these configurations are calculated to determine the most stable adsorption site. The results are shown in Table 1. It is clearly seen that when the TM atom is located on T_WS_ or T_WSe_, the system has the lowest energy, indicating that the top of W atom is the most stable adsorption site. Hence, all the following calculations of electronic structures and magnetic properties are based on this configuration. Table 2 lists the calculated results including the bond length (d_W-S_, d_W-Se_, and d_TM-S(Se)_), the height difference (∆*h*) between the upper layer S(Se) atom and the TM atom, the total magnetic moment M_T_, the local magnetic moment M_L_ of TM atom, and MAE. Obviously, the structure parameters of d_W-S_ and d_W-Se_ are different from that of the primitive Janus WSSe. For the case that the TM atoms are adsorbed on the S-side of WSSe, the d_W-S_ is elongated by comparing with that in primitive Janus WSSe (2.41 Å), whereas the d_W-Se_ is almost kept same (2.54 Å). Similarly, this behavior occurs in the case that TM atoms are adsorbed on the Se-side, where the d_W-Se_ is also expanded. This is because that the covalent interaction between the TM atoms and the neighboring S(Se) atoms weakens the coupling between W and S(Se) and then leads to the extension of the W-S(Se) bond. In addition, the d_TM-S(Se)_ and the ∆h for the different adsorption surface are distinct. They exhibit the smaller value for the S adsorbing surface, which is owing to the stronger electronegativity for the S atom, as revealed in Fig. 1b.

**Table 1:** The total energy for different configurations

TM	S side (eV)			Se side (eV)		
	T_WS_	H_S_	T_S_	T_WSe_	T_Se_	H_Se_
Co	−375.365	−375.134	−373.631	−374.820	−373.484	−373.477
Fe	−376.865	−376.584	−374.894	−376.386	−374.373	−375.394
Mn	−376.816	−376.635	−376.276	−376.269	−375.028	−376.252
Cr	−377.387	−377.177	−376.909	−377.089	−376.797	−373.477
V	−376.623	−376.155	−375.459	−376.007	−375.275	−375.894

**Table 2:** The calculated results including the bond length (d_W-S_, d_W-Se_, and d_TM-S(Se)_), the height difference (∆*h*), the total magnetic moment M_T_, the local magnetic moment M_L_ of TM adatom, and MAE

TM	Side	d_W-S_ (Å)	d_W-Se_ (Å)	d_TM-S(Se)_ (Å)	∆*h*(Å)	Μ_L_ (μB)	M_T_ (μB)	MAE (meV)
Co	S	2.51	2.52	2.12	1.24	0.92	1.00	0.79
	Se	2.41	2.66	2.22	1.37	0.93	1.00	2.68
Fe	S	2.51	2.53	2.16	1.21	1.83	2.00	2.66
	Se	2.42	2.67	2.24	1.36	1.88	2.00	−0.95
Mn	S	2.47	2.54	2.21	1.36	2.73	3.00	3.88
	Se	2.43	2.61	2.31	1.45	2.78	3.00	3.33
Cr	S	2.47	2.54	2.41	1.82	4.80	6.00	−2.72
	Se	2.42	2.57	2.60	1.91	4.86	6.00	−0.57
V	S	2.50	2.53	2.31	1.70	2.90	5.00	−4.19
	Se	2.42	2.62	2.42	1.84	2.98	5.00	−2.76

In the following, we focus on the magnetic behavior of Janus WSSe after the adsorption of TM atoms. As shown in Table 2, the distinguished magnetism for the different configurations is observed. A maximal M_T_ of 6 μB is obtained in Cr-adsorbed system. Interestingly, different adsorption surfaces do not cause an obvious difference in the M_T_, albeit there is a relatively big difference in the M_L_. The calculated M_L_ are 0.92, 1.83, 2.73, 4.80, and 2.90 μB on the S surface, and 0.93, 1.88, 2.78, 4.86, and 2.98 μB on the Se surface for Co, Fe, Mn, Cr, and V adatoms, respectively. Notably, the M_L_ on the S surface is always smaller than that on the Se surface for each kind of TM adatom, indicating the stronger-induced magnetism in Janus WSSe for the case of S adsorbing surface.

**Fig. 3:**
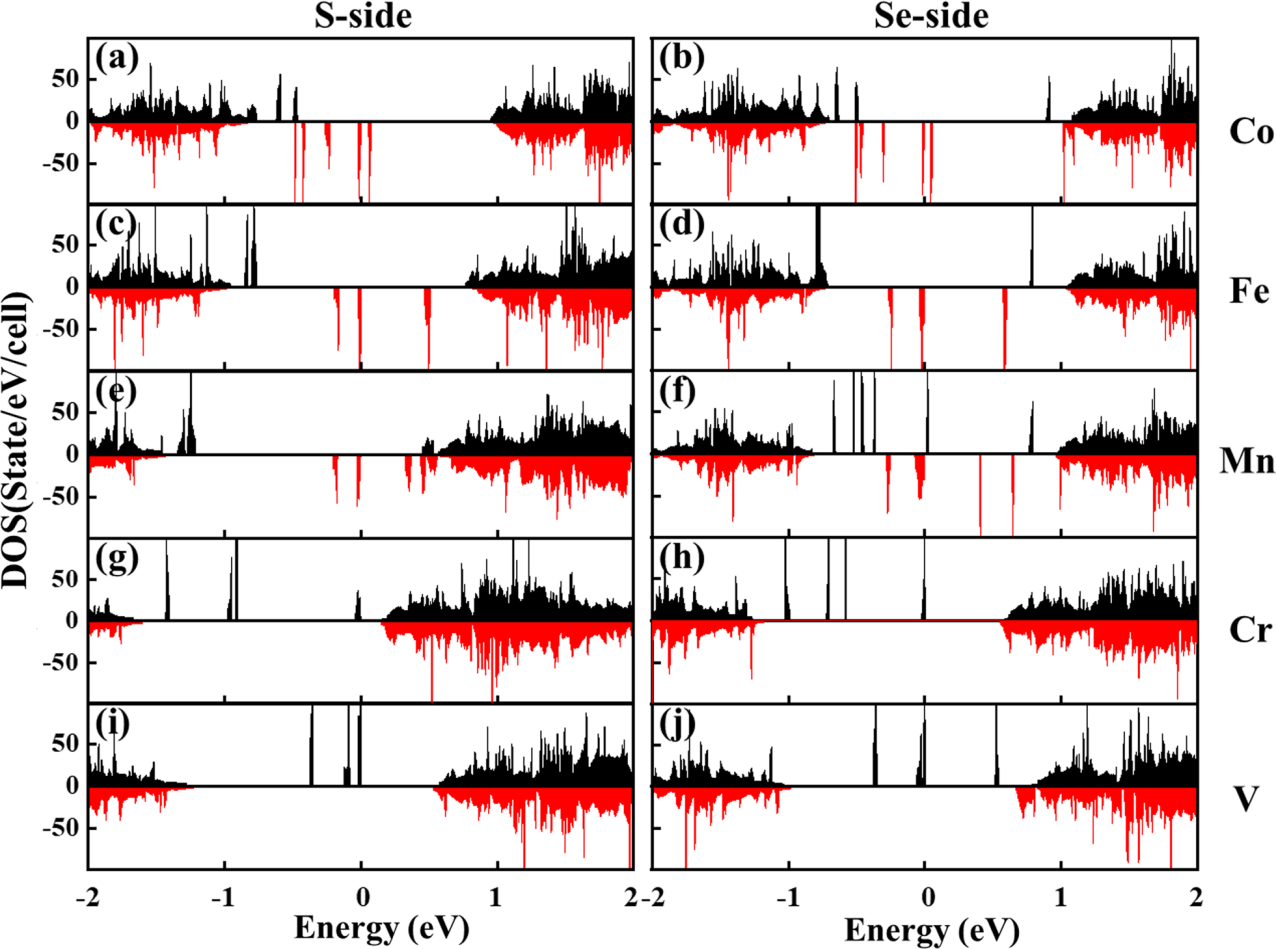
Spin-polarized total DOS of the different TM atom-adsorbed WSSe monolayer. **a**, **b** Co; **c**, **d** Fe; **e**, **f** Mn; **g**, **h** Cr; **i**, **j** V

To gain insight into the magnetic properties of the different systems, the spin-resolved total DOS is calculated with the results shown in Fig. 3. The positive and negative values denote the majority and minority spin channels, respectively, and the Fermi level is set to be zero. The majority and minority spin states in all the systems exhibit asymmetric characterization, confirming the existence of the magnetism. Compared with the DOS of pure Janus WSSe shown in Fig. 1c, some new impurity states appear in the bandgap in all the systems. These impurity states are mainly attributed to the TM-3d states, a small amount of hybridization of the first nearest S-3p or Se-3p states, and the second nearest W-5d states [22]. Due to the localization of TM-3d orbitals, the impurity states show a narrow energy range. Notably, in the case of Co, Fe, and Mn adsorptions, the induced impurity states around Fermi level only distribute in the minority spin channel, demonstrating a 100% spin polarization. Whereas for the other two cases, there are only the majority spin states in the band gap. In addition, due to the influence of the internal electrostatic potential for the different adsorption surfaces, the energy level and intensity of impurity states are slightly different. These results suggest that the magnetic properties strongly depend on the adsorbed element and the adsorbent chalcogen layer.

**Fig. 4:**
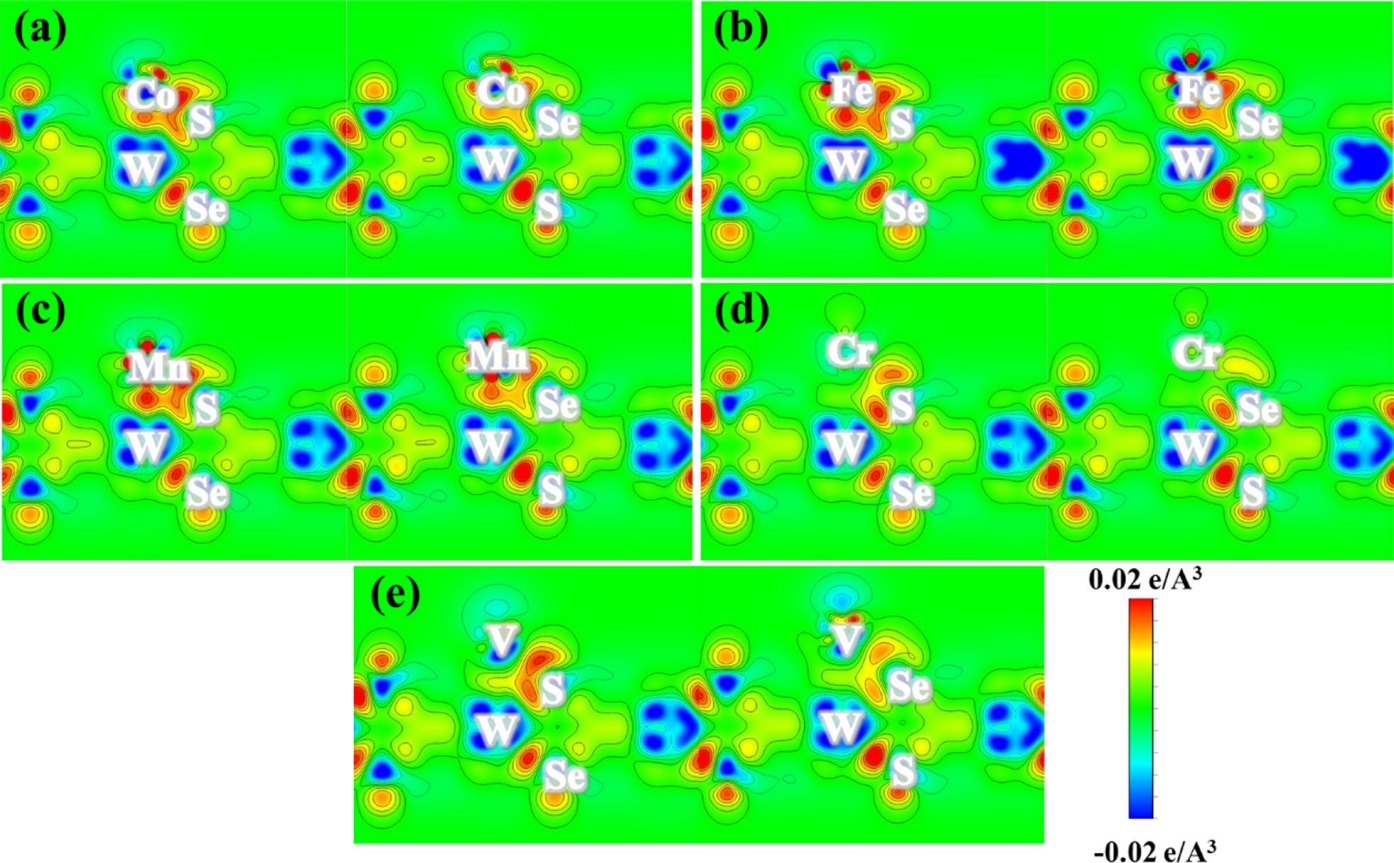
Differential charge densities of different TM atom-adsorbed systems. **a** Co; **b** Fe; **c** Mn; **d** Cr; **e** V

To further reveal the origin of magnetism in different systems, the differential charge densities are calculated. As shown in Fig. 4, there are strong negative differential charge densities around TM atoms and the nearest neighboring chalcogen atoms. While in the middle of the TM-S(Se) bond, significant charge accumulations are observed. This means that the TM atoms and the chalcogen atoms are combined by covalent bonds. It is worth noting that the charge accumulation between TM-S bonds is more evident than that between TM-Se bonds, which indicates the stronger covalent interaction and the shorter bond length. Meanwhile, a small number of charges are accumulated between the TM atom and the lower W atom due to the internal electric field along the z direction. The charge accumulations in the case of Cr and V adsorption are smaller than that in the other cases, which is consistent with the relatively long bond length shown in Table 2. The transfer of charges between the TM atoms and the WSSe layer leads to the decrease of the unpaired electrons in TM atoms, which reduces the magnetic moment of the TM atom on the one hand, and induces the magnetism of the WSSe on the other hand.

The magnetic anisotropy for different systems is investigated as well. The calculated results are shown in Table 2. Positive and negative MAE indicate the vertical and parallel easy magnetization axis of the system, respectively. The Cr- and V-adsorbed systems have the negative MAE, while the Mn- and Co-adsorbed systems show positive MAE, demonstrating that their easy magnetization axis are in-plane and out-of-plane, respectively. Different adsorption surfaces cause slight changes in MAE, but do not give rise to the changes in their easy magnetization axis. Interestingly, the characteristics in the Fe-adsorbed system are completely different. Its easy magnetization axis switches from the in-plane (MAE: −0.95 meV) to the out-of-plane (MAE: 2.66 meV) when the adsorbing surface changes from Se to S.

**Fig. 5:**
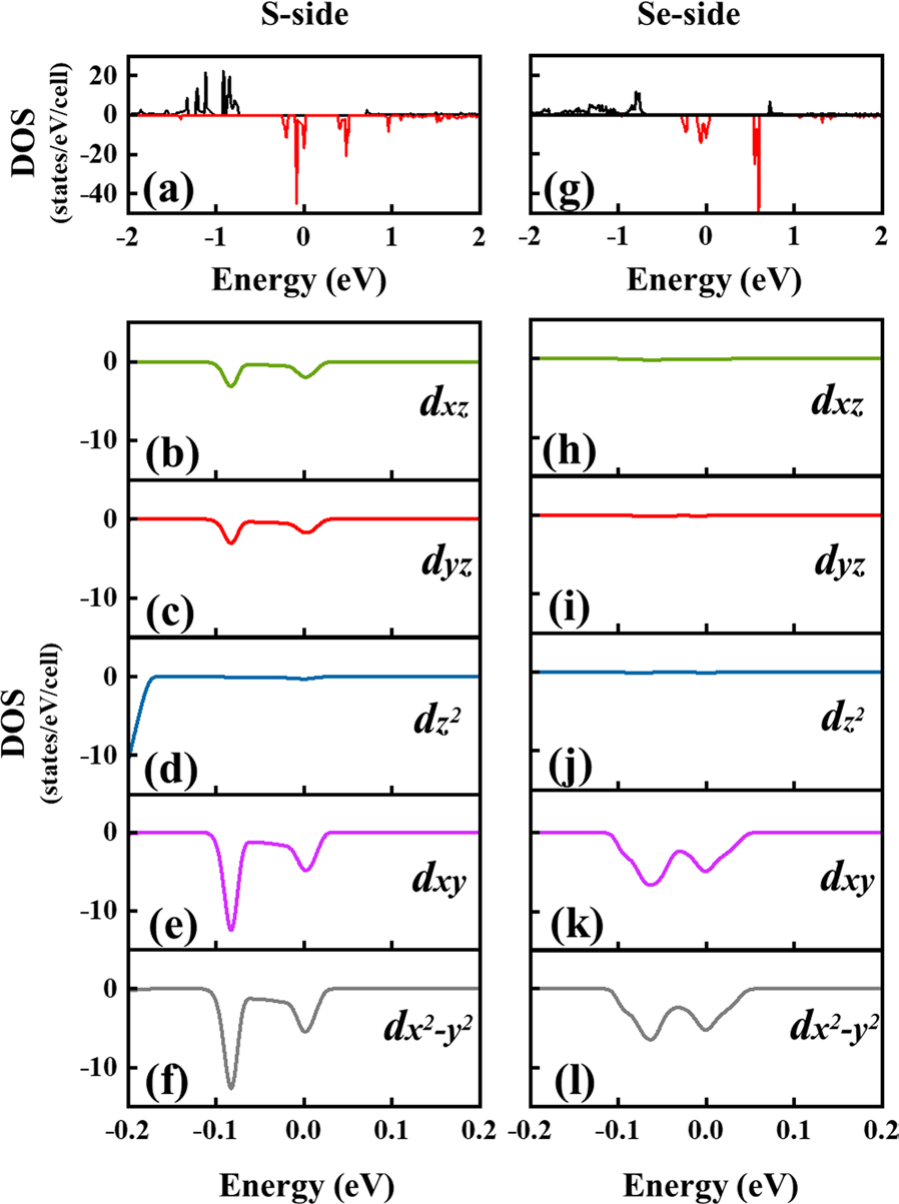
DOS of Fe-adsorbed system with different adsorption surface, **a** on the S adsorption surface; **b** on the Se adsorption surface. **c**–**g** The 3d-orbital decomposed DOS of Fe atom adsorbed on the S surface. **h**–**l** The 3d-orbital decomposed DOS of Fe atom adsorbed on the Se surface

To better understand the mechanism of the changed MAE in Fe-absorbed system, we calculated the Fe-3d orbital-decomposed DOS with the results shown in Fig. 5. According to the second-order perturbation theory [23, 40–42], the MAE arising from the SOC can be approximately formulated as:

1 $$MAE = E_{||} - E_{ \bot } \approx \xi^{2} \mathop \sum \limits_{\mu ,\sigma } \frac{{\mu^{ \downarrow \left( \uparrow \right)} \left| {L_{z} } \right|\sigma^{ \downarrow \left( \uparrow \right)} - \mu^{ \downarrow \left( \uparrow \right)} \left| {L_{x} } \right|\sigma^{ \downarrow \left( \uparrow \right)} }}{{E_{\mu } - E_{\sigma } }}$$

where *σ*↓(↑), *μ*↓(↑) and *Eσ*, *Eμ* denote the eigenstates and eigenvalues of the occupied (unoccupied) states with spin state (↓or↑), respectively; $\xi$ represents the strength of SOC; *Lz* and *Lx* stand for the angular momenta operators. The SOC is regarded as the perturbative term in the Hamiltonian, and the MAE is expressed as the energy difference between the occupied states and the unoccupied states through the coupling of angular momenta *Lz* and *Lx*. In general, MAE is determined by non-zero elements in *Lz* and *Lx* matrices near the Fermi level. As for the same spin states (↓↓or ↑↑), when the occupied and unoccupied states have the same magnetic quantum number *m*, they make a positive contribution to the MAE under the action of the operator *Lz*; whereas when they have different *m*, a negative contribution to the MAE is made through the action of the operator *Lx*. As for the different spin states (↓↑ or ↑↓), the contribution is just the opposite. The non-zero matrix elements include < *xz* | *Lz* | *yz* >  = 1, < *xy* | *L*_*Z*_ | *x*^*2*^*-y*^*2*^ >  = 2, < *z*^2^| *Lx* | *xz, yz* >  = $\sqrt 3$, < *xy* | *Lx* | *xz, yz* >  = 1, < *x*^*2*^*- y*^*2*^ | *Lx* | *xz, yz* >  = 1. In our case, as shown in Fig. 5a, b, only the minority spin states appear near the Fermi level, so it determines the MAE. Ligand-field theory is combination of crystal field theory and molecular orbital theory, which can be used to explain the bonding of coordination compounds and analyze changes in central atomic orbitals [43]. According to ligand-field theory, the C_3v_ symmetry makes the degenerated Fe-3d orbitals split into three kinds of states: single state *a* (d*z*^*2*^, |*m*|= 0), degenerated states *e*_*1*_ (d*yz, *d*xz,* |*m*|= 1) ,and *e*_*2*_ (d*xy, *d*x*^*2*^*−y*^*2*^, |*m*|= 2). As shown in Fig. 5c–g, when Fe is adsorbed on the S surface, the DOS mainly includes the *dxz, dyz, dxy*, and *dx*^*2*^*-y*^*2*^ minority spin states, and a significant positive contribution to MAE comes from the spin-conservation term < *xz* | *Lz* | *yz* >  = 1 and < *xy* | *Lz* | *x*^*2*^*-y*^*2*^ >  = 2, whereas the relatively weak negative contribution is from the spin-conservation term < *xy* | *Lx* | *xz, yz* >  = 1, < *x*^*2*^*- y*^*2*^ | *Lx* | *xz, yz* >  = 1. As a result, a positive MAE of 2.66 meV is achieved. As for the case of Fe adsorbed on Se surface, the *dxz* and *dyz* minority spin states dramatically reduces, and as the result, the MAE reduces to -0.95 meV owing to the significant decrease of the positive contribution term < *xz* | *Lz* | *yz* >.

Since the electronic and magnetic properties of the system depend on different adsorbed elements and different adsorbed surfaces, achieving an accurate detection of TM atoms deposition in experiments is essentially critical and could be a challenge for MAE engineering. In view of this, a spin-polarized scanning tunneling microscope (STM) equipped with a magnetic tip can be engaged to gain insight on the spin states near the adsorption sites and X-ray magnetic circular dichroism (XMCD) measurements can also be carried out to investigate the magnetic moment information and MAE of TM atoms on Janus material [44].

## Conclusions

In this work, we systematically studied the structures and the magnetic properties of different TM atom-adsorbed WSSe frameworks by the first-principles calculations. The pristine Janus WSSe monolayer shows the different potential energies on S and Se surfaces due to the broken mirror symmetry along the *Z* direction. Meanwhile, it is non-magnetic in nature. The adsorbed configurations have the lowest energy when TM atom adsorbs on T_WS_ or T_WSe_, indicating the most stable adsorption site. All the adsorbed systems exhibit magnetism. Their magnetism strongly depends on the adsorbed elements and the adsorbent chalcogens. The maximal M_T_ of 6 μB is obtained in the Cr-adsorbed system. Different adsorption surfaces do not cause an obvious difference in M_T_; however, there was a relatively big difference in M_L_. The M_L_ for the S surface adsorption is always smaller than that for the Se surface adsorption due to the stronger electrostatic potential, revealing the stronger-induced magnetism. The differential charge densities reveal that the magnetism of the system is attributed to the covalent interaction and the charge transfer between TM atoms and WSSe. In addition, different adsorption surfaces do not result in the changes of the easy magnetization axis in Cr-, V-, Mn-, and Co-adsorbed systems. However, as for the Fe-adsorbed system, the easy magnetization axis switches from the in-plane to the out-of-plane when the adsorption surface changes from Se to S surface. It is found that the strong coupling between the minority states d*xy, *d*x*^*2*^* − y*^*2*^ and d*xz, *d*yz* on the S surface contribute to the positive MAE, while the dramatically reduced *dxz* and d*yz* minority spin states on the Se surface leads to the negative MAE. Since adsorbed atoms is an effective method to induce magnetism in two-dimensional systems, it offers insightful guidance to the preparation of magnetic Janus TMDC and design the novel 2D spintronic devices.

## Data Availability

All data are fully available without restriction.

## Abbreviations

TM: Transition metal
TMDCs: Transition metal dichalcogenides
SOC: Spin–orbit coupling
DOS: Density of states
MAE: Magnetic anisotropy energy
STM: Scanning tunneling microscope
XMCD: X-ray magnetic circular dichroism
